# Trait and State-Dependent Risk Attitude of Monkeys Measured in a Single-Option Response Task

**DOI:** 10.3389/fnins.2019.00816

**Published:** 2019-08-07

**Authors:** Atsushi Fujimoto, Takafumi Minamimoto

**Affiliations:** Department of Functional Brain Imaging, National Institute of Radiological Sciences, National Institutes for Quantum and Radiological Science and Technology, Chiba, Japan

**Keywords:** risk attitude, subjective value, decision-making, monkeys, economic models

## Abstract

Humans and animals show diverse preferences for risks (“trait-like” risk attitude) and shift their preference depending on the state or current needs (“state-dependent” risk attitude). For a better understanding of the neural mechanisms underlying risk-sensitive decisions, useful animal models have been required. Here we examined the risk attitude of three male monkeys in a single-option response task, in which an instrumental lever-release was required to obtain a chance of reward. In each trial, reward condition, either deterministic (100% of 1, 2, 3, and 4 drops of juice) or probabilistic (25, 50, 75, and 100% of 4-drop juice) was randomly selected and assigned by a unique visual cue, allowing the monkeys to evaluate the forthcoming reward. The subjective value of the reward was inferred from their performance. Model-based analysis incorporating known economic models revealed non-linear probability distortion in monkeys; unlike previous studies, they showed a simple convex or concave probability distortion curve. The direction of risk preference was consistent between early and late phases of the testing period, suggesting that our observation reflected the trait-like risk attitude of monkeys, at least under the current experimental setting. Regardless of the baseline risk preference, all monkeys showed an enhancement of risk preference in a session according to the satiation level (i.e., state-dependent risk attitude). Our results suggest that, without choice or cognitive demand, monkeys show naturalistic risk attitude – diverse and flexible like humans. Our novel approach may provide a useful animal model of risk-sensitive decisions, facilitating the investigation of the neural mechanisms of decision-making under risk.

## Introduction

In an uncertain environment, one’s preference toward risk biases one’s decisions. Imagine that your friend encouraged you to buy an unlisted stock of a business venture. If you are a conservative person, you may pass on the opportunity to avoid the risk (i.e., risk-averse). However, if you are an adventurous person, you may buy the stock regardless of the risk (i.e., risk-prone). As such, inherent individual risk preference is diverse and determines the basic tendency to take (or not to take) a risky option (“trait-like” risk attitude) (Weber et al., 2002; Huettel et al., 2006; Tobler et al., 2008). In addition, the risk attitude is changeable depending on internal contexts; if you need to make money right away, you may buy the risky stock irrespective of your character (“state-dependent” risk attitude) (Caraco et al., 1980; Stephens and Krebs, 1986; McNamara and Houston, 1992).

Past studies measured the risk preference of human subjects in economic tasks, in which subjects repeatedly made choices between a risky option and a safe option, and mathematical models have been proposed to capture the choice decisions of subjects. The most influential model, prospect theory, assumes a distortion of probabilities and provides better explanation of the non-normative choice pattern of human subjects than the expected utility theory does (Kahneman, 1979; Tversky and Kahneman, 1992; Prelec, 1998; Gonzalez and Wu, 1999). Calculation of the subjective value based on distorted probability is conceptually analogous to the assumption of the finance theory that calculates the subjective value with the mean–variance model (Markowitz, 1952; Levy and Markowitz, 1979; Tobler et al., 2009). These studies revealed various risk preferences of human subjects, and further facilitated research to find the neural correlates of trait-like risk attitude by coupling with brain imaging techniques (Tom et al., 2007; Takahashi et al., 2010; Gilaie-Dotan et al., 2014). Such an economic approach has also been applied to some animal studies using a liquid reward as an alternative of a monetary reward, and they consistently reported non-linear probability distortion of monkeys just like humans (Stauffer et al., 2015; Chen and Stuphorn, 2018).

Although economic approaches began to elucidate the mechanisms of risk-sensitive decisions across species, direct application of economic tasks to animals may pose limitations; for example, the cognitive capacity (e.g., working memory) of animals is not comparable to that of humans, but is largely limited to adaptation to their ecological niche (Krebs et al., 1977; Stevens et al., 2005; Elmore et al., 2011). Such disparity may enforce extra task-demands on animals even in physically identical task settings (Pearson et al., 2010; Blanchard et al., 2013). Another problem is that making repeated choices among available options is an unfamiliar setting for animals considering their feeding ecology, in which they typically make a cost–benefit decision on a single prey (i.e., non-choice decisions) (Krebs et al., 1977; Kacelnik et al., 2011; Hayden and Walton, 2014). As recently suggested, such non-choice decisions recruit distinct brain circuits to that for two-option choices (Kolling et al., 2012; Shenhav et al., 2016). Moreover, some studies using human subjects emphasized that humans showed distorted risk preference in the task without choice (Tobler et al., 2008; Levy et al., 2011). Hence, from an ethological perspective, it is worthwhile to test the risk preference of monkeys in a non-choice decision paradigm.

In this study, we aimed to assess the naturalistic risk attitude of monkeys by minimizing undesirable task demands. We adopted a non-choice, instrumental lever-release task, in which a visual cue revealed the size and probability of forthcoming reward condition as being either deterministic or probabilistic. The basic setting of this task was shown to be useful for inferring monkeys’ evaluation of a certain reward value (e.g., reward size) based on their performance (Minamimoto et al., 2009). The inference has been formulated and applied in many studies (Bouret and Richmond, 2015; Eldridge et al., 2016; Nagai et al., 2016; Fujimoto et al., 2019), and can be extended to temporal discounting and workload discounting using the same basic task structure (Minamimoto et al., 2009, 2012). Here, we implemented well-known economic models to assess the trait-like and state-dependent risk attitude of monkeys in a quantitative manner (Stauffer et al., 2015; Chen and Stuphorn, 2018). Our results may fill the gap between human and monkey studies using economic tasks, thus providing a useful animal model to investigate the neural basis of risk-sensitive decision-making.

## Materials and Methods

### Subjects

Three male macaque monkeys (*Macaca mulatta*, monkeys ST and KY, 5.3 kg and 6.8 kg; *Macaca fuscata*, monkey HI, 7.6 kg) were used. All experimental procedures were approved by the Animal Care and Use Committee of the National Institutes for Quantum and Radiological Science and Technology and were in accordance with the guidelines published in the NIH Guide for the Care and Use of Laboratory Animals.

### Behavioral Task

The monkeys squatted on a primate chair inside a dark, sound-attenuated, and electrically shielded room. A touch-sensitive lever was mounted on the chair. Visual stimuli were displayed on a computer video monitor in front of the animal. Behavioral control and data acquisition were performed using a real-time experimentation system (REX) (Hays et al., 1982). Presentation software was used to display visual stimuli (Neurobehavioral Systems Inc., Berkeley, CA, United States).

**FIGURE 1:**
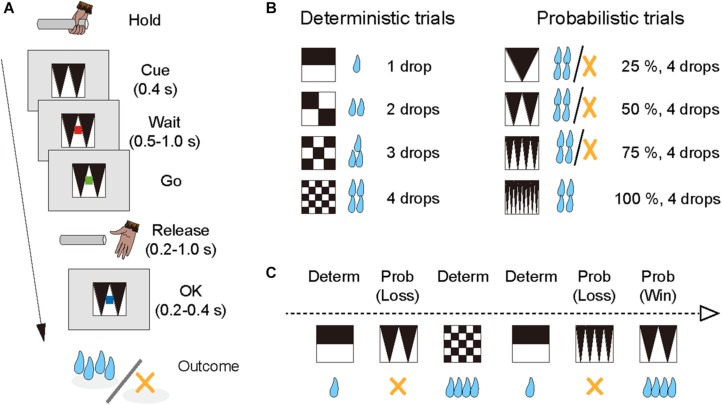
Single-option response task. **(A)** Sequence of a trial. **(B)** Cue sets. Left: cue stimuli that predict deterministic reward delivery (deterministic trials). Right: cue stimuli that predict probabilistic reward delivery (probabilistic trials). **(C)** An example of trial series. Deterministic and probabilistic trials were intermingled in a session.

The monkeys performed the single-option response task (Figure 1A). In each trial, the monkey had the same requirement to obtain liquid rewards. A trial began when a monkey gripped a lever. A visual cue and a red spot appeared sequentially, with a 0.4 s interval, at the center of the monitor. After a variable interval (0.5–1.5 s), the central spot turned to green (“go” signal), and the monkey had to release the lever within the reaction time (RT) window (0.2–1.0 s). If the monkey released the lever correctly, the spot turned to blue (0.2–0.4 s), and then a reward was delivered in accordance with the visual cue. The next trial began following an inter-trial interval (ITI, 1.5 s). When trials were performed incorrectly, they were terminated immediately (all visual stimuli disappeared), and the next trial began with the same reward condition following the ITI. There were two types of errors: premature lever releases (lever releases before or no later than 0.2 s after the appearance of the go signal, named “early errors”) and failures to release the lever within 1.0 s after the appearance of the go signal (named “late errors”).

The combination of reward size and its probability was informed by the visual cue (grayscale images) at the beginning of each trial; four cues were used for the deterministic trials and the other four for the probabilistic trials (Figure 1B). In the deterministic trials, the size of the reward (1, 2, 3, or 4 drops) was chosen randomly, and the reward probability was fixed at 100%. In the probabilistic trials, the size of the reward was fixed at 4 drops and the probability of the reward (25, 50, 75, or 100%) was chosen randomly. Thus, the expected value was matched across the two conditions. The training schedule was as follows. Prior to the experiment with the single-option response task, all monkeys had been trained to perform color discrimination trials in a cued multi-trial reward schedule task for >1 month. Next, the monkeys were trained in the deterministic trials for 3 weeks, and subsequently in the probabilistic trials for 3 weeks, respectively (“separate” phase). Finally, the monkeys were tested under the condition in which deterministic and probabilistic trials were intermingled, and the test ran for >6 weeks (“mixed” phase; Figure 1C). The data obtained during the mixed phase (43, 53, and 41 sessions for monkeys ST, KY, and HI, respectively) were analyzed in the current study. The number of trials in a session was 1,338 ± 79 trials for monkey ST, 1,206 ± 300 trials for monkey KY, and 1,384 ± 109 trials for monkey HI, and the amount of reward intake in a session was 325 ± 20 ml for monkey ST, 286 ± 75 ml for monkey KY, and 327 ± 38 ml for monkey HI (mean ± SD).

### Experimental Design and Statistical Analysis

All statistical analyses and model fitting were performed using R statistical software. We analyzed the error rate and RT. The error rate was calculated by dividing the total number of errors (the sum of early and later errors) by the total number of trials in a session. We reported the average error rate across sessions and the standard error of the mean (SEM). RT was defined as the duration from a “go” signal to the time point of lever release in a correct trial.

As previously shown, the error rate in the same paradigm with deterministic reward has an inverse relationship to the subjective value (inverse function, Minamimoto et al., 2009). To infer the subjective reward value in each monkey, we used a modified version of the inverse function:

(1) $$E=\frac{c}{V+b}$$

where *E* and *V* represented the error rate and the subjective value, while *c* and *b* were free parameters that represented the reward sensitivity of monkeys. We confirmed that this model fitted well with the error rates in deterministic trials of the training session, where (*V)* corresponded to the reward size (1, 2, 3, and 4 drops; *R*^2^ > 0.86). We extended this model to infer the subjective reward value of probabilistic trials using three models: *GW*, *Prelec*, and *mean–variance models* (see below). For each monkey, parameters *c* and *b* were first determined using the best-fit of the inverse function (Eq. 1) to the error rate in the deterministic trials. These parameters were then applied to Eq. (1), which integrated one of the three subjective value models as *V* and then was fitted to the error rates in the probabilistic trials.

#### GW Model

According to Gonzalez and Wu (1999), probability weighting function, *w*(*p*), was formulated as below:

(2) $$w\,\left(p\right)=\frac{\delta \,p^{\gamma }}{\delta \,p^{\gamma }+{\left(1-p\right)}^{\gamma }}$$

where *p* represents the probability of winning a reward (25, 50, 75, and 100%), and γ and δ are free parameters that control the curvature and elevation of the function, respectively. This model yields non-linear probability weighting function, although it allows monotonic increase/decrease of probability weighting when γ = 1. Subjective value *V* was then calculated by multiplying the reward magnitude *m* (4 drops) and subjective probability *w*(*p*) in accordance with the prospect theory (Kahneman, 1979; Tversky and Kahneman, 1992).

(3) V=m×w⁢(p)

#### Prelec Model

According to Prelec (1998), the probability weighting function was formulated as below:

(4) $$w\,\left(p\right)=e^{\left(-\beta \,{\left(-\ln\left(p\right)\right)}^{\alpha }\right)}$$

where α and β are free parameters that control the curvature and elevation of the function, respectively. For the one-parameter Prelec model, β is fixed at 1; this function yields an inverted S-shape in α > 1, while it yields S-shape in α < 1, with inflection point (*p* = *w*(*p*)) around *p* = 1/*e*. We defined the subjective values with Eqs (3) and (4).

#### Mean–Variance Model

According to financial theory, the subjective value is determined by combining the expected value (EV) and variance risk (Var) (Markowitz, 1952; Levy and Markowitz, 1979). First, EV and Var are calculated as follows:

(5)  ⁢EV=m×p

(6) $$\text{Var}={\left(\left(m-\text{EV}\right)\times p\right)}^{2}+{\left(\left(0-\text{EV}\right)\times \left(1-p\right)\right)}^{2}$$

Then, the subjective value is defined as:

(7) V=EV+Var×ε

where ε is a free parameter that describes a bonus by the variance risk.

The model fittings were performed using the “*optim*” function implemented in R software. Standard error of estimated parameter was calculated by means of the Hessian matrix at the function. The goodness of fit was assessed with the *R*^2^ value and Akaike Information Criteria (AIC) (Akaike, 1973), which is calculated as follows:

(8) AIC=-2⁢log⁡L+2⁢k

where *L* is the maximum likelihood of the model and *k* is the number of free parameters in the model. Smaller AIC values indicated a better model fit to the data. A likelihood ratio test was used to compare GW models. The *p*-value was obtained by the parametric bootstrapping method (*n* = 10,000).

The effect of the satiation level on risk attitude was assessed using a measure of accumulated reward level (Minamimoto et al., 2009). Satiation level (*S*) was defined as the normalized liquid intake that is the ratio between the amount of total reward delivered up to time *t*, *R*_cum_(*t*), and the total amount of reward delivered in the entire session, *R*_cum_Max:

(9) $$S=\frac{R_{\text{cum}}\,\left(t\right)}{R_{\text{cum}}\,\text{Max}}$$

The effect of the history of previous reward was also assessed by logistic regression analysis:

(10) $$P=\beta_{1}\,R+\beta_{2}\,S+\beta_{3}\,P\,R+e$$

where *P* is the performance (i.e., correct or error), *R* is the reward size, *S* is the satiation level, *PR* is the reward size in the previous trial, β are the regression coefficients, and *e* is a constant.

## Results

### Risk Preference in Three Monkeys

**FIGURE 2:**
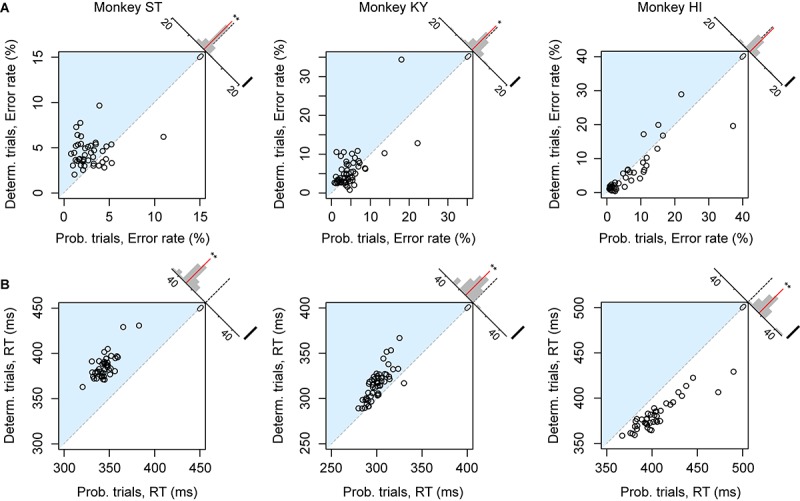
Risk-induced behavioral bias. **(A)** Error rate of each session. Error rates in probabilistic trials (abscissa) and in deterministic trials (ordinate) in each session are plotted for monkeys ST (left), KY (center), and HI (right). The plots in the blue shaded areas indicate risk-prone sessions. Histograms on the right shoulder of panels show the distribution of the distance between each plot and the identity line. Red lines indicate the average of the distance. Asterisks indicate significant difference from zero (^∗∗^*p* < 0.01, ^*^p < 0.05, rank-sum test). **(B)** Reaction time (RT) of each session. Schemas of the figures are the same as in **A**.

The error rate and RT were the two main behavioral measures of the monkeys’ valuation of the current task; the more reward value is expected, the less the subjects make errors and the faster they respond (Minamimoto et al., 2009; Nagai et al., 2016; Fujimoto et al., 2019). We first compared the overall error rate and RT between deterministic (1, 2, or 3 drops) and probabilistic trials (25, 50, or 75%) in each session separately. For this analysis, we excluded the trials of which the expected value was 4 drops (and the probability was 100%) to focus on the effect of risk. Although expected values were equivalent between the two trial types, motivation of monkey ST appeared to be higher in probabilistic trials; the overall error rate in the deterministic trials was significantly higher than that in the probabilistic trials (*n* = 43, *p* < 0.01, rank-sum test; Figure 2A, left), and RT in the deterministic trials was significantly longer than in the probabilistic trials (*n* = 43, *p* < 0.01, rank-sum test, Figure 2B, left). These results indicated a risk-prone tendency of this monkey, which was consistent across sessions. Monkey KY also showed a risk-prone tendency; the error rate and RT were significantly larger and longer in the deterministic trials (error rate, *n* = 53, *p* = 0.049; RT, *n* = 53, *p* < 0.01; Figures 2A,B, middle column). Monkey HI, on the other hand, displayed the opposite pattern; the error rate and RT tended to be larger and longer in the probabilistic trials (error rate, *n* = 41, *p* = 0.54; RT, *n* = 41, *p* < 0.01; Figures 2A,B, right column), indicating a risk-averse tendency of this monkey. These results demonstrate that our task allowed us to characterize the individual risk preference of monkeys as a consistent behavioral bias across sessions, which was not uniform across the monkeys examined.

**FIGURE 3:**
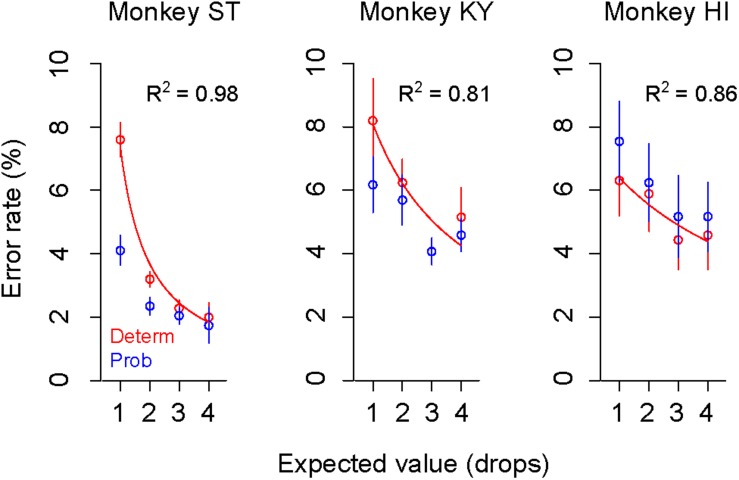
Change of error rate by reward-expected value and risk. Error rates (mean ± SEM) in deterministic (red) and probabilistic trials (blue) are plotted as a function of expected values for monkey ST (left), KY (center), and HI (right). The best-fit inverse function (red) is superimposed on the plots (ST: *c* = 7.3, *b* = –1.8; KY: *c* = 26.9, *b* = 2.3, HI: *c* = 41.9, *b* = 5.6) with the goodness of fit (*R*^2^) on each panel.

As we reported previously, the error rate in the deterministic trials varied depending on the reward size, with higher error rates for smaller reward (Figure 3, plots in red), the relation of which was well explained by an inverse function (Eq. 1, *R*^2^ > 0.80) (Minamimoto et al., 2009; Nagai et al., 2016). The error rate in the probabilistic trials also reflected the expected value of reward; however, they were lower (monkeys ST and KY) or higher (monkey HI) than those in deterministic trials for the corresponding expected value (Figure 3, plots in blue). Three-way ANOVA (expected value: 1, 2, 3, and 4 drops × trial type: deterministic or probabilistic × Monkey) revealed a significant main effect of the expected value [*F*_(__1__,__1088__)_ = 39.6, *p* < 0.01] and a significant interaction of the trial type and monkey [*F*_(__1__,__1088__)_ = 4.9, *p* = 0.027], suggesting the effects of reward expectation and individual risk preference on the subjective valuation of probabilistic rewards.

### Simulations With Parsimonious Models

To describe the relationship between error rate and reward probability, we used a modified version of the inverse function with the subjective value of probabilistic reward (i.e., subjective-value model). To estimate the subjective valuation of monkeys, we employed the probability-weighting function developed by Gonzalez and Wu (1999) (“GW model,” Eq. 2), a prospect-theory model that is widely used to describe non-linear probability distortion measured in economic tasks. Because both probabilistic and deterministic trials were tested in the same sessions, we used the same monkey-specific parameters *c* and *b* in the inverse functions to explain the error rates in two trial types (see the section “Materials and Methods”).

**FIGURE 4:**
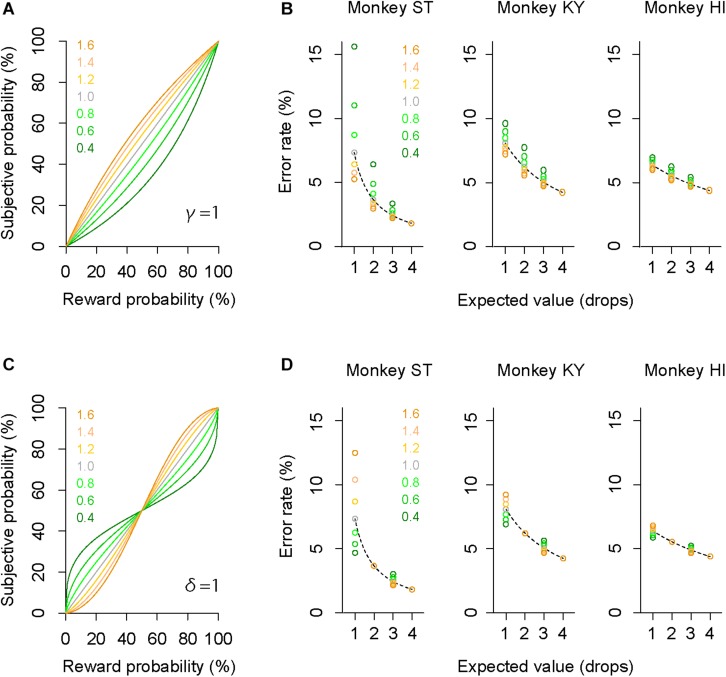
Simulation of error rates in probabilistic trials. **(A)** Simulated probability-weighting function with partial GW model with fixed γ. Colors indicate the value of parameter δ used for the simulation. **(B)** Simulated error rate in the probabilistic trials with partial GW model (γ = 1) for monkey ST (left), KY (center), and HI (right). As a reference, a best-fit inverse function to error rate in deterministic trials (dashed gray curve) is shown for each monkey. **(C,D)** Simulated probability-weighting function **(C)** and error rate in the probabilistic trials **(D)** with partial GW model with fixed δ (δ = 1). Colors indicate the value of parameter γ used for the simulation. Schemas of the figures are the same as in **A** and **B**.

The GW model implements two free parameters: γ and δ, control curvature and elevation of function, respectively. First, we simulated how each parameter modifies the probability-weighting function and the error rate by using parsimonious models (“partial GW models”), which incorporate one free parameter. When γ in the GW model was fixed [GW (δ| γ = 1)], the probability-weighting function became concave when δ > 1, while it became convex when δ < 1 (Figure 4A). The error rate in the probabilistic trials then simply rose or fell compared to that in the deterministic trials (Figure 4B). When δ in the GW model was fixed [GW (γ| δ = 1)], on the other hand, the function became S-shaped when γ > 1, while it became inverted S-shaped when γ < 1 (Figure 4C). Under this condition, the error rates in the two trial types crossed each other; when γ < 1, for instance, the error rate in 25% trials was lower than in 1-drop trials and that in 75% trials was higher than in 3-drop trials (Figure 4D). Because the data demonstrated simple reduction (monkeys ST and KY) or elevation (monkey HI) of error rate by imposing risk (Figure 3), the simulation suggests that the partial GW model with fixed γ [GW (δ| γ = 1)] may explain the probability distortion of monkeys.

### Modeling Individual Risk Preference Reflecting Trait-Like Risk Attitude

**FIGURE 5:**
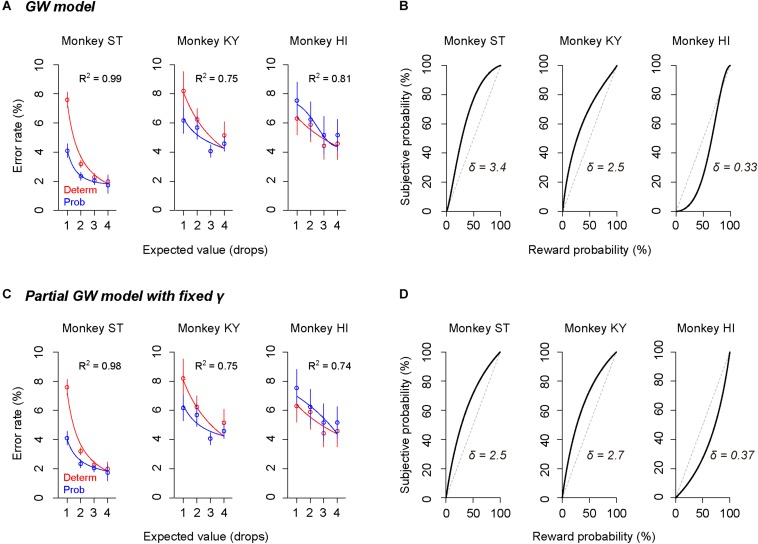
Validation of the full and partial GW models. **(A,C)** Error rate and best-fit function of subjective-value models in the deterministic trials (red) and in the probabilistic trials (blue) for monkeys ST (left), KY (center), and HI (right). Red curve shows the best-fit inverse function for each monkey. Blue curve shows the best-fit function of the subjective-value model with the GW model [GW (δ, γ)] **(A)** or with the partial GW model with fixed γ [GW (δ | γ = 1)] **(C)**. **(B,D)** Best-fit probability-weighting function for each monkey. Probability-weighting function is calculated in the GW model **(B)** or in the partial GW model with fixed γ **(D)**, and value of estimated parameter δ is shown in each panel. Dashed line indicates the identity line where subjective probability and reward probability are indifferent.

The subjective-value model implementing the GW model [GW (γ, δ)] well explained the error rate in the probabilistic trials for all monkeys (*R*^2^ > 0.75, Figure 5A). As predicted in the simulation, the best-fit probability-weighting function with the GW model showed a simple convex or concave pattern (Figure 5B), demonstrating overweighting of reward probability (monkeys ST and KY) and underweighting of reward probability (monkey HI) in subjective valuation of the probabilistic reward. This result suggests risk-prone tendency of monkeys ST and KY and risk-averse tendency of monkey HI, as demonstrated in Figure 2. Then, to validate the parsimonious model, we tested whether the partial GW model with fixed γ [GW (δ| γ = 1), Figure 4A] also fits the data. As expected, the subjective-value model implementing the partial GW model with fixed γ well described the error rate in the probabilistic trials for all monkeys (*R*^2^ > 0.74, Figure 5C). The best-fit probability-weighting function and estimated parameter δ (Figure 5D) was comparable to those estimated by the full GW model. In contrast, the partial GW model with fixed δ or the simple GW model with fixed γ and δ did not provide good fits to the error rate in the probabilistic trials [GW (γ| δ = 1) and GW (γ = 1, δ = 1), Table 1]. The partial GW model, GW (δ| γ = 1), explained the data significantly better than the simple GW model in all monkeys (*p* < 0.05, likelihood ratio test), suggesting that unfixed parameter δ is essential and sufficient for explaining the individual risk preference of monkeys measured in the single-option response task. We also tested whether the subjective-value model (the inverse function fusing the partial GW model with fixed γ), which incorporated three free parameters *c*, *b*, and δ, fits the error rate in both trial types. The model again fitted well with the data for all monkeys (*R*^2^ > 0.81), suggesting the robustness of the modified inverse function in the current task.

**TABLE 1:** Comparison of GW models.

**Model**	**Estimated parameters**			**AIC**		
	**Monkey**			**Monkey**		
	**ST**	**KY**	**HI**	**ST**	**KY**	**HI**
GW (γ, δ)	1.3, 3.4	0.89, 2.5	1.7, 0.33	−2.5^*^	12.4	12.4
GW (δ | γ = 1)	2.5	2.7	0.37	0.98^*^	10.5^*^	11.8^*^
GW (γ | δ = 1)	0.36	0.49	2.69	16.3	16.6	13.8
GW (γ = 1, δ = 1)				19.9	16.2	13.4

^*^The significant better fits than GW (γ = 1, δ = 1) (*p* < 0.05; likelihood ratio test).

As shown in Figure 2, the risk in reward outcome biased error rate and RT in the same direction, and the direction of bias was roughly consistent during the testing period. Given that what we modeled reflected the trait-like risk attitude of monkeys, the direction of risk preference (i.e., risk-prone or risk-averse), in other words, a convex or concave probability weighting pattern, should be stable over a longer time period. To confirm the stability of individual risk preference, we separately calculated δ in the partial GW model for the early (e.g., #1–20 sessions) and late testing sessions (e.g., #21–40 sessions) for each monkey. As expected, risk preference was consistent over the sessions; monkeys ST and KY showed high δ (>1) either in early or late sessions (ST early: 2.8 ± 1.0, ST late: 2.3 ± 0.6; KY early: 1.3 ± 0.8, KY late: 3.2 ± 0.9, mean ± SEM), while monkey HI consistently showed low δ (<1) between the two periods (early: 0.25 ± 0.43, late: 0.51 ± 0.14). These results suggested that we modeled the trait-like risk attitude of the monkeys.

### Convex/Concave Probability Distortion Was Not Model-Specific

**FIGURE 6:**
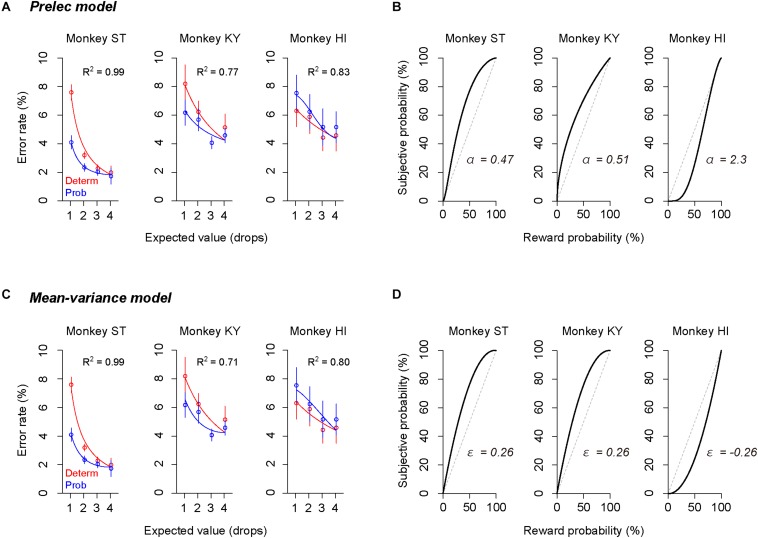
Validation of Prelec model and mean–variance models. **(A,C)** Error rate and best-fit functions of subjective-value models for each monkey. Blue curve shows best-fit function of the subjective-value model implementing the Prelec model **(A)** or the mean–variance model **(C)** (blue), respectively. Schemas of the figures are the same as in Figures 5A,C. **(B,D)** Best-fit probability-weighting function calculated by the Prelec model **(B)** or by the mean–variance model **(D)**. Schemas of the figures are the same as in Figures 5B,D.

The error rate was also well explained by other subjective-value models that incorporated the Prelec model (Eq. 4, *R*^2^ > 0.77, Figure 6A) or mean–variance model (Eq. 7, *R*^2^ > 0.71, Figure 6C), which also assume non-linear probability distortion (Markowitz, 1952; Levy and Markowitz, 1979; Prelec, 1998). The best-fit probability-weighting function calculated by the Prelec model (Figure 6B) or mean–variance model (Figure 6D) showed the convex or concave pattern that was comparable to that calculated by the full or partial GW model (Figures 5B,D). Thus, the individual risk preference assessed in the single-option response task can be modeled reasonably well by the economic models with a free parameter focusing on the elevation. The goodness of fit (AIC) and parameters estimated are summarized in Table 2.

**TABLE 2:** Summary of goodness of fit and estimated parameters.

**Model**	**Estimated parameters**			**AIC**		
	**Monkey**			**Monkey**		
	**ST**	**KY**	**HI**	**ST**	**KY**	**HI**
GW (δ | γ = 1)	2.5	2.7	0.37	0.98	10.5	11.8
Prelec (β, α)	1.6, 0.47	1.1, 0.51	1.4, 2.3	–2.1	12.1	12.1
Mean variance (ε)	0.26	0.26	−0.26	–3.1	11.1	10.8

### Assessing State-Dependent Risk Attitude Within a Session

**FIGURE 7:**
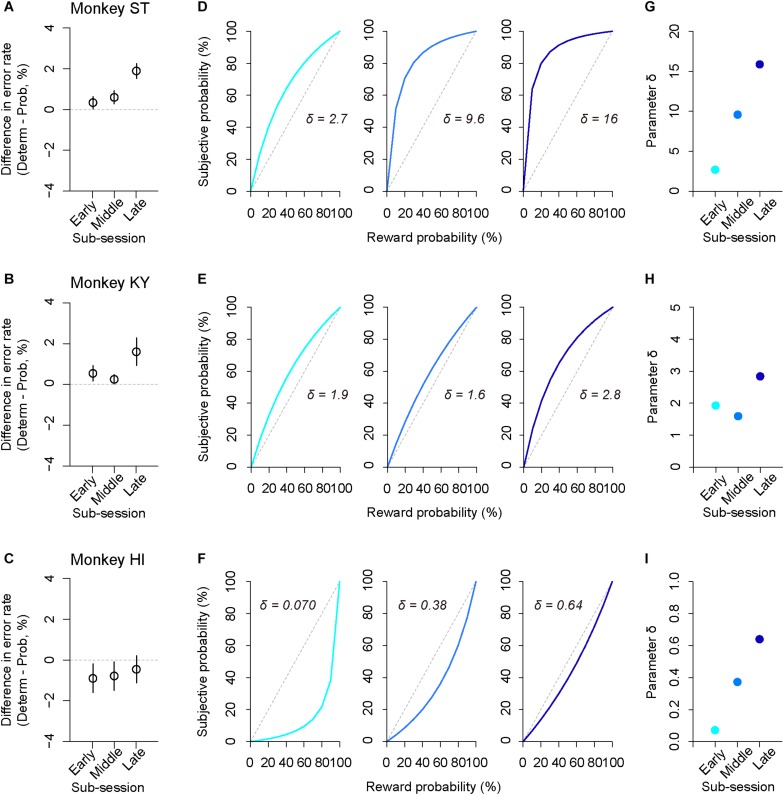
Satiation effect on risk attitude. **(A–C)** Difference in error rate by trial type. The error rate in the probabilistic trials was subtracted from that in the deterministic trials for each sub-session (early, middle, late). Panels are for monkeys ST **(A)**, KY **(B)**, and HI **(C)**. **(D–F)** Shifts of probability-weighting functions (Eq. 2) according to satiation for each monkey. The best-fit function for the data of each sub-session (left: 0–0.5, center: 0.25–0.75, right: 0.5–1.0, satiation level) is displayed. **(G–I)** Parameter δ is plotted for each sub-session and for each monkey. Colors are the same as in **D–F**.

In addition to trait-like risk attitude, physiological drive state can influence risk attitudes; for example, thirsty monkeys became more risk averse (Yamada et al., 2013). To examine the effect of satiation on risk attitude, we analyzed the error rate in the sub-parts of a session according to reward accumulation (satiation level: 0–0.5, 0.25–0.75, 0.5–1.0; see the section “Materials and Methods”). We found that the difference in error rate between deterministic and probabilistic trials varied depending on the satiation level [one-way repeated measures ANOVAs, main effect of satiation level, *F*_(__1__,__409__)_ = 5.9, *p* = 0.015, Figures 7A–C]. The satiation level also affected RT; the difference in RTs between the two conditions increased according to satiation [main effect of satiation level, *F*_(__1__,__409__)_ = 17, *p* < 0.01].

The satiation effect on risk attitude was further assessed by the modeling approach; we fitted the subjective-value model implementing the partial GW model with fixed γ to the error rate in the probabilistic trials and extracted the best-fit parameter δ from the probability-weighting function for each sub-session (Figures 7D–F). We found that parameter δ tended to increase in the latter sub-sessions for all monkeys; the risk-proneness of monkeys ST and KY was evident in the early period and was enhanced thereafter, while monkey HI exhibited weaker risk-averseness as the session progressed and became nearly risk-neutral in the last sub-session (Figures 7G–I). In contrast, the direction of risk attitude was unchanged over a session; δ was always >1 in monkeys ST and KY, whereas it was always <1 in monkey HI. These results demonstrated a state-dependent risk attitude in monkeys; that is, the risk preference gets stronger according to satiation.

### Partial Effects of Reward History on Performance

**FIGURE 8:**
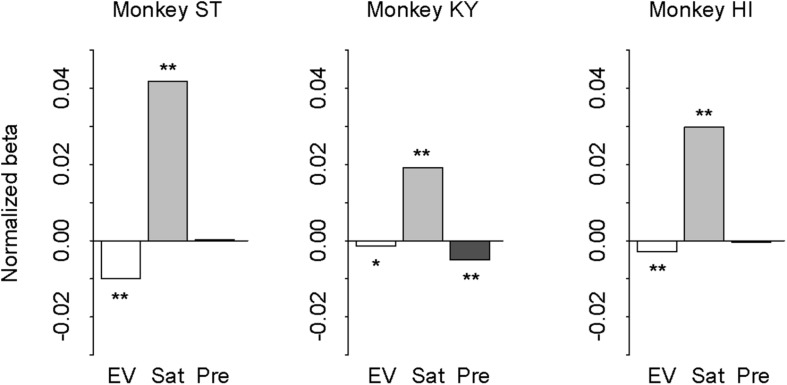
Effect of expected value, satiation, and reward history on performance. Results of logistic regression for monkey ST (left), KY (center), and HI (right). Bars indicate the correlation coefficient (normalized beta) of expected value (white), satiation level (gray), and previous reward (black), respectively. Asterisks indicate statistically significant difference from zero (^∗∗^*p* < 0.05, ^*^p < 0.10 with Bonferroni correction).

In our task design, the subjective value of probabilistic reward was associated with the cue but was independent from trial sequence or history. However, monkeys could take local contextual reward information into account for the reward expectation that may influence the performance (i.e., correct or error). In other words, the differences in error rate between the deterministic and probabilistic trials could arise from the effect of reward history. If so, the effect should be parallel with the risk preferences of the three monkeys. To address this possibility, we performed logistic regression analysis with three regressors: expected value (1, 2, 3, or 4 drops), satiation level (0–1), and previous reward (0, 1, 2, 3, or 4 drops). Expected value and satiation level significantly contributed to the performance for all monkeys (*p* < 0.05 with Bonferroni correction; Figure 8). The previous reward, on the other hand, affected only the performance of monkey KY (*p* < 0.01), but not the other two (*p* > 0.10, Figure 8). This pattern of individual differences was unrelated to that of risk preference or state-dependent change among the three monkeys. Thus, the effect of reward history was apparently limited and did not correlate with individual risk attitude in our experimental condition.

## Discussion

In the present study, monkeys’ risk attitude was assessed by a single-option response task, in which the subjective value of a probabilistic reward was inferred from their performance. To the best of our knowledge, this is the first study to examine risk preference of monkeys in a non-choice paradigm. Model-based analysis revealed non-linear probability distortion and diverse risk preference among three monkeys. The subjective probability weighting of monkeys was well explained by economic models and showed a simple convex/concave pattern over testing sessions. Regardless of baseline risk preference, all monkeys showed an increase in risk preference as satiation increased in a session. The current results thus highlighted the trait-like and state-dependent risk attitude of monkeys in non-choice decisions.

Past studies demonstrated that monkeys show non-linear probability distortion using economic tasks (Stauffer et al., 2015; Chen and Stuphorn, 2018). The present study replicated this in the single-option response task that imposed no choice demand. The basic structure of the current task was shown to be useful to infer the valuation of monkeys when reward size or cost was varied (Minamimoto et al., 2009, 2012; Bouret and Richmond, 2015; Eldridge et al., 2016; Nagai et al., 2016; Fujimoto et al., 2019). By implementing known economic models, the present study extended this basic model to infer the subjective reward value of probabilistic reward. Our monkeys demonstrated a diverse preference for the risk; two monkeys showed risk-prone, and one showed risk-averse. This seems to reflect the trait-like risk attitude of monkeys because their risk preferences were consistent across sessions. Their performance in probabilistic trials was well demonstrated by a subjective-value model incorporating a non-linear probability weighting function (Markowitz, 1952; Levy and Markowitz, 1979; Prelec, 1998; Gonzalez and Wu, 1999), and thus the results were largely consistent with the above literature despite differences in task structures and measures of subjective valuations. Our results also suggest that economic models are generalizable for describing the probability distortion in non-choice, ecological decisions (Hayden and Walton, 2014; Pearson et al., 2014).

Unlike the previous studies, our monkeys showed a simple convex or concave probability distortion, and that pattern was well explained by a parsimonious GW model in which one free parameter concerning the elevation of function was adopted. On the above studies using economic tasks, all monkeys tested showed inverted S-shaped probability distortion (i.e., risk-seeking for low probability and risk-aversion for high probability) and was well-explained by Prelec’s function with α < 1, while the same model failed to explain the monkeys’ performance in the current study. Such a stereotypical pattern observed in the previous studies may arise from excessive task demand in economic tasks; the cognitive load due to choice demand could diminish sensitivity to the difference in the reward probability and result in the inverted S-shape probability distortion. Indeed, recent studies showed that manipulation in task structure (e.g., trial sequence) of economic tasks affected monkeys’ inverted S-shape probability distortion, potentially due to contamination of reward history (Farashahi et al., 2018; Ferrari-Toniolo et al., 2019). Importantly, the effect of reward history was limited in our paradigm, and hence did not account for the observed individual risk preference. Therefore, the discrepancy could be attributed solely to the task design concerning the ecological decision situation.

As a genetic kinship, humans and monkeys share a large number of cognitive traits. However, because monkeys learn the option value through their experience, a task structure *per se* would largely influence their task performance and therefore hamper a straightforward interpretation by investigators (Real, 1991). For example, Blanchard et al. (2013) demonstrated that monkeys did not care about the length of the delay period after reward delivery, and that had led to misunderstanding by preceding researchers about the temporal-discounting ability of monkeys. Similarly, economic tasks could contain undesirable confoundings, such as working memory, inhibitory control, and value comparison, which may affect decision strategy and obscure natural behavioral traits (Stephens and Krebs, 1986; Elmore et al., 2011; Blanchard et al., 2014; Hayden and Walton, 2014). The current study eliminated such undesirable confounding effects by adopting a non-choice decision in the task. In fact, our monkeys quickly learned to perform the single-option response task (<1 month), while it usually takes several months for monkeys to learn to perform two-option choices. Unlike using choice tasks, diverse individual differences in trait-like risk attitudes were seen in our monkeys, as observed in human studies (Tom et al., 2007; Tobler et al., 2008; Takahashi et al., 2010; Gilaie-Dotan et al., 2014), and therefore the current task may provide a better opportunity to assess the naturalistic risk attitude of monkeys.

Adapting risk attitude based on current needs is vital for maximizing fitness in an uncertain environment (Stephens and Krebs, 1986). Human studies showed that subjects flexibly modulate risk attitude based on required points or “wealth level” even during a single experimental session (Symmonds et al., 2011; Kolling et al., 2014; Fujimoto and Takahashi, 2016; Juechems et al., 2017). Yamada et al. (2013) directly demonstrated the relationship between risk preference and satiety by monitoring the blood osmolality level within a session in macaque monkeys, which is a physiological form of “wealth level.” Consistently, our monkeys showed enhancement of risk-prone tendency (ST and KY) or suppression of risk-aversion (HI) according to reward accumulation, and our model-based analysis successfully described the satiation effect. Of note, the increase of risk preference reflects state-dependent risk attitude, because it occurred irrespective of baseline risk preference. This change of risk preference within a session is not attributable to the reward history effect, which was limited in the monkeys. Importantly, human studies suggested that state-dependent modulation of risk attitude was not accounted for by change of the physiological state itself either (Symmonds et al., 2011; Kolling et al., 2014; Fujimoto and Takahashi, 2016). Hence, the current approach successfully quantified the trait-like and state-dependent risk attitude of monkeys within one task, suggesting a useful model of risk-sensitive decision for translational research.

What causes the inconsistent risk preference across animals still remains unclear. Probably the most well-known factors that lead to differences in risk attitude in humans are gender and age (Walker et al., 2017). However, they are unlikely to have a role in the current study because we solely used adult male monkeys. Another possible cause is social rank (Davis et al., 2009), but the contribution of this factor is unknown because we have not tested the social relationship of our monkeys. Future study should validate the exact cause of individual risk preference by employing a larger cohort of animals.

Past studies reported that the trait risk attitude correlated with individual differences in monoamine systems (Berridge and Waterhouse, 2003; Roiser et al., 2009; Takahashi et al., 2010), brain structures (Gilaie-Dotan et al., 2014; Leong et al., 2016), and activity patterns (Kuhnen and Knutson, 2005; Huettel et al., 2006; Preuschoff et al., 2008; Levy et al., 2010) of human subjects. However, the neural substrates of individual risk preference in monkeys are largely unknown. Our behavioral assessment, which successfully demonstrated diverse risk attitude in monkeys with single free parameter (δ), may provide an excellent opportunity to explore the neural basis of individual risk preference, as the animal model allows us to measure neural activities directly, and to use neural modulation techniques (cf., Nagai et al., 2016). One of the potential applications is the study of gambling disorder (GD), which is considered to be a dysfunction of risk-sensitive decision (Hodgins et al., 2011; American Psychiatric Association [APA], 2013). Indeed, we recently showed that GD patients had deficits not only in trait-like risk attitude but also in state-dependent risk attitude (Fujimoto et al., 2017). Therefore, future study should identify the neural substrates of both trait-like and state-dependent risk attitude in monkeys, providing therapeutic targets for GD patients.

One of the limitations of the current study was the small sample size. We thus could not address the mechanism behind individual differences in risk attitude. Another limitation was that we used only one reward size for probabilistic trials (4 drops); modifying the range of reward size may influence monkeys’ risk attitude. Further validation with a larger cohort and/or broader reward environments will be needed to generalize our findings and identify other factors that influence the risk attitude of monkeys.

In conclusion, our approach based on economics and behavioral ecology illustrates the trait-like and state-dependent risk attitude of monkeys. Because our model-based analysis employed well-known functions from past human studies, the current animal model may accelerate translational research to determine neural mechanisms underlying risk-sensitive decision-making.

## Data Availability

All datasets generated for this study are included in the manuscript and/or the supplementary files.

## Ethics Statement

All experimental procedures were approved by the Animal Care and Use Committee of the National Institutes for Quantum and Radiological Science and Technology and were in accordance with the guidelines published in the NIH Guide for the Care and Use of Laboratory Animals.

## Author Contributions

AF designed and performed the research, analyzed the data, and wrote the manuscript. TM designed the research and edited the manuscript.

## Conflict of Interest Statement

The authors declare that the research was conducted in the absence of any commercial or financial relationships that could be construed as a potential conflict of interest.
